# Coagulation Parameters and Risk of Progressive Hemorrhagic Injury after Traumatic Brain Injury: A Systematic Review and Meta-Analysis

**DOI:** 10.1155/2015/261825

**Published:** 2015-09-17

**Authors:** Danfeng Zhang, Shun Gong, Hai Jin, Junyu Wang, Ping Sheng, Wei Zou, Yan Dong, Lijun Hou

**Affiliations:** Department of Neurosurgery, Changzheng Hospital, Second Military Medical University, Shanghai 200003, China

## Abstract

Intracranial hemorrhage (ICH) after traumatic brain injury (TBI) commonly increases in size and coagulopathy has been implicated in such progression. Our aim is to perform a meta-analysis to assess their relationship. Cochrane library, PubMed, and EMBASE were searched for literatures. Pooled effect sizes and 95% confidential intervals (CIs) were calculated using random-effects model. We included six studies, involving 1700 participants with 540 progressive hemorrhagic injuries (PHIs). Our findings indicate that PT, D-dimer level, and INR value are positively associated with the risk of PHI. Higher level of PLT and Fg seemed to suggest a lower risk of PHI. Among these parameters, higher D-dimer level and INR value would possess more powerful strength in predicting PHI.

## 1. Introduction

Traumatic brain injury (TBI) remains the leading cause of death after trauma, often leading to long term physical and neuropsychiatric deficits [1–6]. Although some damage to the brain occurs in the initial of injury, secondary brain damage due to ongoing intracranial bleeding and brain swelling is an eminent and potentially avoidable cause of morbidity and mortality. Of many potential secondary processes, progressive hemorrhagic injury (PHI) is one of the most important and devastating issues [7].

PHI is generally defined as the appearance of new hemorrhage lesion or evident expansion of previous hematoma [8–13], with an incidence ranging from 6% to 67% [8–26]. Traditionally, PHI is diagnosed by repeated CT scanning before irreversible deterioration occurs. Nevertheless, practices of some trauma centers turned out to be time-consuming and costly [20]. Therefore, addressing risk factors closely associated with PHI would be of great avail. Several factors, such as male gender, low initial level of consciousness, older age, and presence of the spot sign on CT angiography and coagulopathy, have been implicated as impact factors of intracranial hemorrhage (ICH) progression [8, 27–29]. Coagulopathy, traditionally diagnosed by routine laboratory tests such as international normalized ratio (INR), activated partial thromboplastin time (APTT), and platelet count (PLT), is common in TBI and may be a potential prognostic factor.

However, data regarding the association between laboratory tests and PHI have been somewhat inconsistent. Stein and associates indicated that abnormal values of INR, APTT, and PLT were independently correlated with PHI [27]. A study by Oertel and colleagues found that only prolonged PTT was associated with PHI [8], whereas Engström and coworkers reported that only low PLT count was related to PHI [15]. In view of discrepancies among literatures, the purpose of our meta-analysis is to explore the relationship between coagulation tests and PHI to improve evidence-based management of patients with TBI.

## 2. Methods

### 2.1. Search Strategy

Two investigators (Danfeng Zhang and Shun Gong) independently searched Cochrane Library, PubMed, and Embase for pertinent studies examining the association between coagulation parameters and the risk of PHI after TBI. Six main coagulation parameters at admission were investigated, respectively. The search was limited to studies published between 1970 and October 2014. The language was restricted to English. The reference lists of retrieved articles were scrutinized to identify additional relevant studies.

### 2.2. Definition of PHI

We defined PHI as “the appearance of new lesion(s) or a conspicuous increase in the size of hemorrhagic lesion(s), which amounted to no less than a 25% or more increase versus the first post-injury CT scan” [8]. The exact definitions of PHI and cutoff points of each coagulation parameter in included studies are given in Table 1.

### 2.3. Selection Criteria

Studies were considered eligible if they (1) were controlled observational studies; (2) included people suffering TBI; (3) investigated the relationship between abnormal coagulation tests and PHI.

### 2.4. Data Extraction and Quality Assessment

Three authors (Danfeng Zhang, Shun Gong, and Hai Jin) extracted the data in standardized data-collection forms, and 2 authors (Wei Zou and Ping Sheng) assessed the study quality. The Newcastle-Ottawa Scale was used to evaluate the methodological quality [30]. Disagreements among reviewers were resolved by joint review.

### 2.5. Statistical Analysis

We pooled continuous and dichotomous data extracted from pertinent articles, respectively, using the random-effects model. When both data were available in one paper, they were pooled into different aspects of our meta-analysis. Mean difference and odds ratio (OR) were selected as the effect sizes. Correlation coefficient *r*_*s*_ was calculated to assess the association strength for each parameter. A threshold of *P* < 0.1 was used to decide whether heterogeneity was present [31]. In other ways, *P* values were 2 sided, with significance level of 0.05. Heterogeneity was mainly assessed by the *I* ^2^ statistic. We considered low, moderate, and high *I* ^2^ values to be 25%, 50%, and 75%, respectively [32]. Sensitivity analyses were also performed. Stata software (version 12.0; Stata Corporation, College Station, TX) was used for the meta-analysis.

## 3. Results

### 3.1. Literature Search

**Figure 1:**
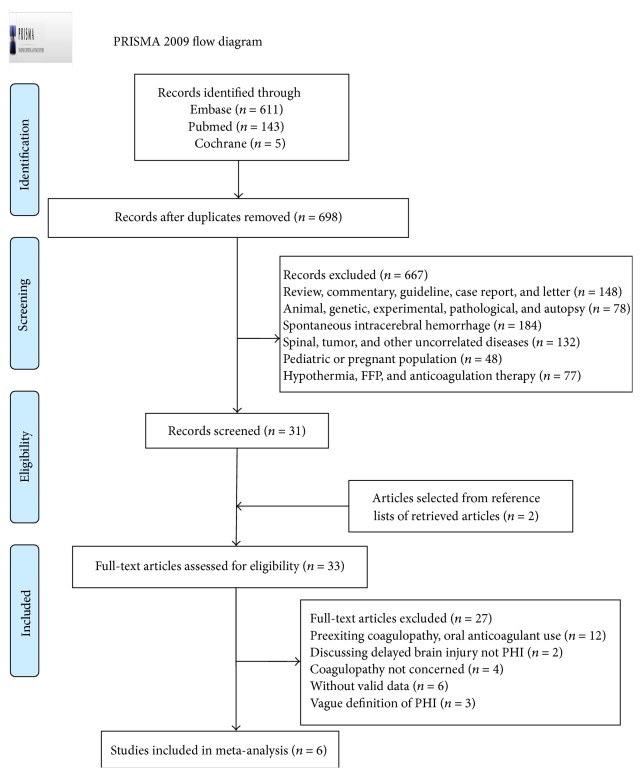
The flow diagram for identifying eligible studies.

The results of study-selection process were shown in Figure 1. Initial search produced 5 studies from Cochrane library, 143 studies from PubMed, and 611 studies from Embase. After exclusion of duplicates and irrelevant studies, 33 potentially eligible studies were selected. After detailed evaluations, 6 studies were selected for final meta-analysis. A manual search of reference lists of these studies yielded 2 eligible studies.

### 3.2. Study Characteristics

A total of 6 studies were included for the meta-analysis, consisting of 4 case-control studies [9–11, 13] and 2 nested case-control studies [8, 12], totaling 1700 subjects with 540 PHIs (Table 2). Subjects in included studies were divided into two groups: PHI-positive group and PHI-negative group. Two studies were conducted in American populations [8, 13] and four in Chinese populations [9–12]. Four studies reported both dichotomous and continuous data for relevant coagulation tests [8–10, 12]. Qualities of included studies were good after assessment (Table 3). Effect sizes and strengths of association between coagulation tests and PHI were summarized in Tables 4 and 5, respectively.

### 3.3. Coagulopathy and Risk of PHI

#### 3.3.1. Coagulation Parameters in PHI versus Non-PHI (Continuous Data)

*PLT and PHI.* Four studies were included to investigate the association between decreased PLT counts and the incidence of PHI following TBI [8, 10, 12, 13]. No significant effect was detected after the meta-analysis (*P* = 0.06).

*PT and PHI.* Two studies were available for meta-analysis with heterogeneous outcomes [8, 10]. The overall result demonstrated no significant effect, with a mean difference of 0.25 (95% CI, −0.60–1.11; *P* = 0.56).

*PTT and PHI.* PTT was tested in four studies designed for the American and the Chinese as well [8, 10, 12, 13]. No positive results were discovered in any study (*P* = 0.92).

*D-Dimer and PHI.* Two studies probed the potential effect of D-dimer [10, 12] on PHI. Significantly increased D-dimer values were found in both studies, which raised no statistically significant result after pooling together, with a mean difference of 34.48 (95% CI, −32.15–101.11; *P* = 0.31).

*Fg and PHI.* We identified three studies [9, 10, 12], two of which showed decreased Fg in patients with PHI. The overall meta-analysis showed a tendency towards Fg reduction in PHI-positive patients: MD of −0.26 (95% CI, −0.39–−0.13; *P* < 0.001), with no heterogeneity (*I* ^2^ = 0, *P* = 0.96).

*INR and PHI.* Two recent studies examining possible relationship between INR and PHI both detected a higher INR in PHI-positive patients [12, 13]. However, gathering extracted data brought out marginal statistical significance [MD: 0.50 (95% CI, −0.09–1.08; *P* = 0.10)].

#### 3.3.2. Prevalence of PHI in Normal Coagulation versus Coagulopathy (Dichotomous Data)

*PLT and PHI.* Dichotomous data for PLT were retrievable from four studies [8–11]. Meta-analysis of included studies suggested statistically significant association between PLT reduction and subsequent PHI, with a pooled OR of 2.76 (95% CI, 1.62–4.70; *P* < 0.001).

*PT and PHI.* Meta-analysis was possible for five studies examining PT values for both groups and reporting dichotomous data [8–12]. Overall, there was a significant increased incidence of PHI with prolonging PT value (OR, 2.69; 95% CI, 1.55–4.66; *P* < 0.001).

*PTT and PHI.* Potential link between PTT and PHI was assessed in four studies [8–11]. The pooled result failed to prove such association (OR, 2.39; 95% CI 0.72–7.95; *P* = 0.15), with a moderate degree of heterogeneity (*I* ^2^ = 70%; *P* = 0.02).

*D-Dimer and PHI.* Three studies hit our search [9–11], demonstrating a significant bound for D-dimer with PHI, with a pooled OR of 16.50 (95% CI, 4.94–55.04; *P* < 0.001).

*Fg and PHI.* Three case-control studies were aggregated to unravel the possible effect of abnormal Fg on the genesis of PHI [9–11]. The summary result favored a strong efficacy of decreased Fg to bring about PHI (OR, 3.44 95% CI, 2.37–4.99; *P* < 0.001).

*INR and PHI.* Meta-analysis was conducted for two studies [11, 13]. General inverse variance method was adopted because of the lack of absolute data in White 2009. Positive associations between INR and PHI were found (OR, 3.70; 95% CI, 1.11–6.29; *P* < 0.001).

### 3.4. Risk Factor Estimates for PHI Associated with Coagulopathy

In order to unravel the connection intensity for each parameter, the correlation coefficient *r*_*s*_ was calculated and assessed through statistical process. As the above, we analyzed both data types, respectively.

#### 3.4.1. Analysis of Continuous Data

Meta-analysis of pertinent studies implied a strong strength for Fg reduction (*r* = −0.13; 95% CI, −0.23–−0.04; *P* < 0.001) and abnormal INR (*r* = 0.47, 95% CI, 0.36–0.59; *P* < 0.001) in predicting PHI. Four studies were retrieved for PLT, with an overall effect size of −0.07. Only marginal statistical significance was detected for D-dimer (*r* = 0.46; *P* = 0.05). Overall analysis gave rise to a nonsignificant effect for PT (*P* = 0.54) and PTT (*P* = 0.97).

#### 3.4.2. Analysis of Dichotomous Data

Powerful impact strength was detected for D-dimer, with summary relative risk of 0.66 (95% CI, 0.44–0.81; *P* < 0.001). Meta-analysis of dichotomous data confirmed moderate statistical association for INR (*r* = 0.35), PT (*r* = 0.33), PLT (*r* = 0.27), and Fg (*r* = 0.30). The pooled result failed to prove significance for PTT (*P* = 0.08).

### 3.5. Sensitivity Analysis

To explore whether our results were influenced by a particular study, we carried out a leave-one-out sensitivity analysis, in which one study at a time was excluded and the remaining ones were analyzed. In sensitivity analysis for PLT, PTT, and Fg, no significantly altered result was shown when excluding studies one by one, whether these indicators were examined as dichotomous variables or continuous variables. As for PT, we found that the study by Oertel et al. [8] accounted for observed heterogeneity. When excluding the study by Oertel et al. [8], the result for PT as a dichotomous variate was more robust (OR = 3.31; 95% CI, 2.47–4.42; *P* < 0.01), with no evidence of heterogeneity (*I* ^2^ = 0; *P* = 0.44). As for D-dimer, it was the study by Tong et al. [10] that brought about the heterogeneity. When excluding the study, the result was more robust (OR = 11.23, 95% CI, 6.30–20.00; *P* < 0.001), with no evidence of heterogeneity (*I* ^2^ = 0; *P* = 0.74).

## 4. Discussion

High prevalence and mortality highlight the importance of timely prognosis of PHI with good sensitivity and specificity. With repeated CT scanning which is time-consuming and costly, addressing the associations between the abnormal coagulation tests and PHI would be of great avail. So whether coagulopathy correlates with PHI occurrence, which laboratory test is meaningful, and which parameter carries the most weight in predicting PHI become burning questions confronting us.

Our findings showed statistically significant positive associations between PT, D-dimer level, INR, and the risk of PHI after TBI. Higher level of PLT and Fg seemed to suggest a lower risk of PHI. Independent PTT seemed to be of no indicative value. As for dichotomous variables, the contributions to PHI were as follows: DD > INR > PT > Fg > PLT. But when examined as continuous variables, the sequence seemed to be INR > DD > Fg > PLT. Meta-analysis of continuous data was perceived to be more meaningful compared with that of dichotomous data because of less conversion steps to correlation coefficient (*r*).

Meanwhile, there were some discrepancies in association strength between dichotomous and continuous data. Contributions of DD and INR were similar, with INR being a little more influential in the analysis of continuous data, while, as a dichotomous variable, DD had the strongest relationship with PHI. The distinction was speculated to derive from different conversion steps, limited included articles, and subjects. As a dichotomous variate, the predicting value for PT was significant, but that was not the case when it was examined as a continuous variate, which might owe to different test methods of included studies. In analysis for PTT as a dichotomous variate, positive association was detected when ignoring Oertel et al. [8] or Tong et al. [10]. When D-dimer was modeled as a dichotomous variate, a robust significance was detected, but we only found a marginally significant positive association for it as a continuous variate. Those too few studies were included and internal nonspecificity might bring the divergence. As for Fg, moderate association with PHI was noticed, whether it was examined as a continuous variable or dichotomous variable. Finally, for INR, powerful association with PHI was detected in both data types.

While the quantitative association between coagulative tests and the risk of PHI has been confirmed, we have to acknowledge that four of our included studies are from China and 2 from America. But this does not significantly mean the incidence of ICH in eastern population is higher than in the counterparts, because of lacking in large prospective epidemic studies in our analysis. Potential bias from population difference should be further evaluated through more large epidemic studies.

No meta-analysis about PHI was published before. There is currently 1 publication that we are aware of that has reviewed the current etiology research available to interpret the predictor and mechanism of PHI [33]. In this paper, relationships between PHI and measurable coagulopathy, while clinically useful, were described to be not one of simple cause and effect. Our findings are in agreement with previous articles by Stein et al. [27], Engström et al. [15], Schnüriger et al. [19], and Allard et al. [16], in which abnormal laboratory test values were independently associated with PHI. In addition, from another perspective, even though PHI was most often diagnosed by the 24-hour CT scan, it might have occurred earlier, preceding and perhaps contributing to the abnormal laboratory tests, which can be regarded as indicators.

Our findings implied that abnormal coagulation tests might indicate occurrence of PHI, which might lead to focused monitoring among TBI patients, and thus save plenty of medical resources. Our interpretation could form the basis for further studies exploring whether correcting these values would prevent PHI and moreover make for subsequent operation. However, there is no strong evidence that correcting laboratorial tests in this situation actually improves outcome. According to Perel et al., there is no reliable evidence from randomized controlled trials to support the effectiveness of hemostatic drugs in reducing mortality or disability in patients with TBI [34], whereas, in another article, tranexamic acid can reduce all-cause mortality in bleeding trauma patients, with no apparent increase in the risk of vascular occlusive events, if given as early as possible and within three hours of injury, as treatment later than this is unlikely to be effective [35]. Therefore further prospective studies are needed to determine the effects of coagulation-associated therapies in patients with TBI. In any case, however, the correction of abnormal values will indisputably ensure safer operation.

As with all meta-analysis, some caveats are in order. Firstly, bias exists because of defects in study design of included studies and meta-analysis itself, such as publication bias, selection bias, confounding bias, and recall bias. Reporting bias might have also occurred, which derived from the disparities in testing method and cutoff point for each parameter in pertinent studies, which, though moderate, might serve as the confounding factors. We tried our best to minimize the bias by excluding those studies with insufficient patient characteristics [8, 19, 21]. Secondly, the limited literature quantity, which partly comes from inaccurate definitions of PHI, is devoted substantially to the heterogeneities between studies. Thirdly, potential indicators in our meta-analysis were all single parameters, while combined parameters, routinely defined as coagulopathy, may be more powerful in predicting PHI. The criteria of coagulopathy have been changed over time. In 2001, the International Society of Thrombosis and Haemostasis (ISTH) simplified the laboratory diagnosis of DIC [36]. However, only one of our included studies had used this score [12]. More studies on relationship between combined parameters and PHI are suggested here. Fourthly, in most studies, blood sample was tested only once upon admission, with the possibility of potential measurement errors. Therefore, dynamic monitoring of coagulation function should be adopted to ensure convincing results. Finally, suggested definition of PHI in most articles only allowed for a 25% or more increase of bleeding in the subsequent CT scan but does not take the absolute bleeding volume into account. As a consequence, comparability between studies or different subjects becomes questionable.

However, our study had strengths in including relatively newer studies with reliable imaging examinations, quantitative analyses, and precise and consistent definition of PHI as we described above. Moreover, we calculated the comparable association strength in predicting PHI after TBI for each parameter for the first time, which was much more clinically meaningful than the association alone. Thus we could assess the prognostic value of these parameters, which might further guide clinical practices.

## 5. Conclusion

Despite the limitations, this meta-analysis has notable clinical and public health implications, which indicate significant inverse associations for PLT level, Fg level, and positive associations for PT, D-dimer level, and INR value in predicting PHI, among which abnormal D-dimer level and INR value were more meaningful. Independent PTT seemed to be meaningless. This study suggests instant test and correction of coagulation parameters at admission to predict PHI and, possibly, better outcome of TBI patients. The focus of our meta-analysis is upon the capability for individual test to predict the presence of PHI after TBI. However, to demonstrate the relationship between coagulopathy and the extent of PHI seems more meaningful, which may be the interest of future studies. Moreover, interrelationships between coagulation tests, which were not discussed here, remain to be unraveled. Eventually, large prospective studies are needed to investigate the underlying pathogenesis better and identify effective therapy to reap the maximum benefits.

## Supplementary Material

Extra information was supplied here to provide detailed methods so that my procedure could be replicated, which contains detailed search strategy, selection criteria, data extraction and quality assessment and statistical analysis as follows.

## Figures and Tables

**Table 1:** Categorization, exposure, continuous data, and definition of PHI of included studies. (Units of measure: PLT, *∗*10^9^/L; PT/PTT, sec; D-dimer, mg/L; Fg, g/L).

Study, the year of publication	Study outcome	Exposure	Cutoff point of every coagulation parameter or coagulopathy	Mean ± SD of PHI group versus non-PHI group	Definition of PHI
Oertel et al., 2002 [8]	PHI	PLT	143	226 ± 74 versus 233 ± 69	PHI was defined as an unambiguous increase in the full film appearance of lesion size; this amounted to a 25% or more increase in at least one dimension of one or more lesions seen on the first postinjury CT scan.
		PT	11.5	12 ± 1.2 versus 12.2 ± 1.5	
		PTT	33.4	26.2 ± 5.4 versus 25.2 ± 4.2	

Sun et al., 2011 [12]	PHI	PLT	—	166.5 ± 53.5 versus 172.4 ± 57.2	PHI was defined as increased appearance of lesion size, which amounted to no less than a 25% increase in one dimension of one or more lesions from the first postinjury CT scan.
		PT	N/A	—	
		PTT	—	25.4 ± 3.5 versus 26.2 ± 5.8	
		D-dimer	—	2.1 ± 2.3 versus 1.3 ± 1.1	
		FIB	—	2.6 ± 1.7 versus 2.8 ± 2.8	
		INR	—	1.9 ± 1.0 versus 1.1 ± 0.5	

Tian et al., 2010 [9]	PHI	PLT	100	—	PHI was defined as the appearance of new lesion(s) or a conspicuous increase in the size of hemorrhagic lesion(s), that is, a 25% increase or more versus the first postinjury CT scan.
		PT	17	—	
		PTT	48	—	
		D-dimer	5	—	
		Fg	2.0	2.33 ± 0.62 versus 2.60 ± 0.65	

White et al., 2009 [13]	PIH	PLT	—	221 ± 84 versus 245 ± 65	Contusion growth was defined as an increase of at least 33% from the initial volume as measured by image analysis on the second CT compared with the baseline CT scan.
		PTT	—	29 ± 5 versus 28 ± 4	
		INR	—	1.4 ± 0.3 versus 1.2 ± 0.2	

Tong et al., 2012 [10]	PIH	PLT	100	168.57 ± 55.22 versus 175.91 ± 51.11	PIH was diagnosed if a patient's repeat CT scan was read as worsening because of new lesions or an increase in the original volume of abnormalities (25% increase in the lesion on the first postinjury CT scan).
		PT	15.5	14.06 ± 1.54 versus 13.39 ± 1.15	
		PTT	39	34.67 ± 6.54 versus 34.46 ± 6.39	
		D-dimer	2.6	80.20 ± 76.75 versus 11.41 ± 14.05	
		Fg	1.8	2.30 ± 1.10 versus 2.56 ± 0.65	

Yuan et al., 2012 [11]	PIH	PLT	150	—	PHI was defined as the appearance of new lesions or a conspicuous increase in the size of hemorrhagic lesions (i.e., a 25% increase or more compared to the first postinjury CT scan).
		PT	14	—	
		PTT	40	—	
		D-dimer	5	—	
		Fg	2.0	—	
		INR	1.2	—	

APTT: activated partial thromboplastin time; D-D: D-dimer; DTICH: delayed traumatic intracerebral/intracranial hemorrhage; Fg/FIB: fibrinogen/fibrin; INR: international normalized ratio; N/A: not available; PHI: progressive hemorrhagic injury; PIH: progressive intracerebral/intracranial hemorrhage; PLT: platelet counts; PT: prothrombin time; SD: standard deviation.

**Table 2:** Characteristics of included studies of coagulation tests and risk of PHI.

Study, the year of publication	Study design	Study population	Number of participants	% men	Age (mean or range) (years)	Exposure	Endpoints (number of cases)	Time of 2nd HCT after admission (mean or range) (hours)
Oertel et al., 2002 [8]	Nested case-control	American	142	81	34 ± 14 (>16)	PLT, PT, and PTT	PHI (60)	<24
Sun et al., 2011 [12]	Nested case-control	Asian (Chinese)	352	68	18–87	INR, PT, APTT, FIB, D-DT, and PLT	PHI (122)	4.9 ± 2.1
Tong et al., 2012 [10]	Case-control	Asian (Chinese)	498	73	44 ± 18	PT, APTT, Fg, PLT, and D-D	PIH (139)	4–6
Yuan et al., 2012 [11]	Case-control	Asian (Chinese)	468	78	47	PT, APTT, Fg, PLT, D-D, and INR	PHI (108)	<24
Tian et al., 2010 [9]	Case-control	Asian (Chinese)	194	79	43.9 ± 15.4	PT, APTT, Fg, PLT, D-D, and INR	PHI (81)	<24
White et al., 2009 [13]	Case-control	American	46	82	38 (11–78)	PLT, PTT, and INR	PHI (30)	12 ± 6.5

APTT: activated partial thromboplastin time; D-D: D-dimer; DTICH: delayed traumatic intracerebral/intracranial hemorrhage; Fg/FIB: fibrinogen/fibrin; INR: international normalized ratio; N/A: not available; PHI: progressive hemorrhagic injury; PIH: progressive intracerebral/intracranial hemorrhage; PLT: platelet counts; PT: prothrombin time; SD: standard deviation.

**Table 3:** Quality scores of case-control studies using Newcastle-Ottawa Scale (maximum score of 9).

Reference	Selection				Comparability	Outcome			
	Adequate definition of cases	Representativeness of cases	Selection of controls	Definition of controls	Comparability on the basis of the design or analysis	Assessment of exposure	Same method of ascertainment for cases and controls	Nonresponse rate (<20%)	Overall
Oertel et al., 2002 [8]	1	1	0	1	1	1	1	1	7
Sun et al., 2011 [12]	1	1	0	1	1	1	1	1	7
Tong et al., 2012 [10]	1	1	0	1	2	1	1	1	8
Yuan et al., 2012 [11]	1	1	0	1	2	1	1	1	8
Tian et al., 2010 [9]	1	1	0	1	2	1	1	1	8
White et al., 2009 [13]	1	1	0	1	2	1	1	1	8

0 = “no,” “unable to determine,” or “not available.”

**Table 4:** Effect of coagulopathy on PHI after traumatic brain injury.

Data type	Coagulation tests	Number of studies	Population size	*P* for test	Heterogeneity (*I* ^2^)	MD/OR (95% CI)
Continuous	PLT	4	1003	0.06	0	−7.24 [−14.77, 0.28]
	PT	2	630	0.56	90	0.25 [−0.60, 1.11]
	PTT	4	1026	0.92	33	0.05 [−0.85, 0.94]
	D-dimer	2	850	0.31	99	34.48 [−32.15, 101.11]
	Fg	3	1044	<0.001	0	−0.26 [−0.39, −0.13]
	INR	2	398	0.10	96	0.50 [−0.09, 1.08]

Dichotomous	PLT	4	1267	<0.001	35	2.76 [1.62, 4.70]
	PT	5	1644	<0.001	70	2.69 [1.55, 4.66]
	PTT	4	1289	0.15	70	2.39 [0.72, 7.95]
	D-dimer	3	1160	<0.001	63	16.50 [4.94, 55.04]
	Fg	3	1145	<0.001	0	3.44 [2.37, 4.99]
	INR	2	512	<0.001	0	3.70 [1.11–6.29]

APTT: activated partial thromboplastin time; Fg: fibrinogen/fibrin; INR: international normalized ratio; PHI: progressive hemorrhagic injury; PLT: platelet counts; PT: prothrombin time; CI: confidence interval; MD: mean difference; OR: odds ratio.

**Table 5:** Summary of the strength of coagulation tests in predicting PHI after traumatic brain injury.

Data type	Coagulation test	Number of studies	Population size	Fisher's *z*	Weighted *r*	Range of effect size *r*
Continuous	PLT	4	1003	−0.07^*∗*^	−0.06	−0.13–−0.003
	PT	2	630	0.10	—	—
	PTT	4	1026	0	—	—
	D-dimer	2	850	0.50^*∗*^	0.46	0.01–0.75
	Fg	3	1044	−0.13^*∗*^	−0.13	−0.23–−0.04
	INR	2	398	0.52^*∗*^	0.47	0.36–0.59

Dichotomous	PLT	4	1267	0.28^*∗*^	0.27	0.10–0.43
	PT	5	1654	0.34^*∗*^	0.33	0.16–0.48
	PTT	4	1289	0.29	—	—
	D-dimer	3	1160	0.80^*∗*^	0.66	0.44–0.81
	Fg	3	1145	0.31^*∗*^	0.30	0.22–0.38
	INR	2	512	0.37^*∗*^	0.35	0.27–0.42

APTT: activated partial thromboplastin time; Fg: fibrinogen/fibrin; INR: international normalized ratio; PHI: progressive hemorrhagic injury; PLT: platelet counts; PT: prothrombin time; ^*∗*^ *P* < 0.05.
