# Supplementary material for: Coagulation Parameters and Risk of Progressive Hemorrhagic Injury after Traumatic Brain Injury: A Systematic Review and Meta-Analysis
Source: Biomed Res Int. 2015 Sep 17;2015:261825. doi: 10.1155/2015/261825 (PMC4589576; doi:10.1155/2015/261825)
Supplement: Supplementary file 1 — Extra information was supplied here to provide detailed methods so that my procedure could be replicated, which contains detailed search strategy, selection criteria, data extraction and quality assessment and statistical analysis as follows. [file 261825.f1.docx]

**ADDITIONAL MATERIAL**

**Coagulation Parameters and Risk of Progressive Hemorrhagic Injury after Traumatic Brain Injury: AMeta-Analysis.**

Danfeng Zhang, MD; Shun Gong, MD; Hai Jin, MD; Junyu Wang, MD;Ping Sheng, MD; Wei Zhou, MS; Yan Dong, MD; LijunHou, MD

**Additional Methods**

*Search Strategy*

Our meta-analysis was conducted in accordance with the Preferred Reporting Items for Systematic Reviews and Meta-analysis (PRISMA) statement.^1^ Two investigators (D.F.Z. and S.G.) independently searched Cochrane Library, Pubmed and Embase for pertinent studies examining the associations between coagulation tests and the risk of PHI. The specific search strategies are as follows:

**Pubmed：**

#1.((head or craniu* or crania* or cerebra* or cerebru* or capitis or brain* or forebrain* or skull* or hemispher* or intra-cran* or inter-cran*) and (injury or injuries or trauma or traumas or traumatic or damag* or wound* or fracture* or contusion*))

#2.((delayed OR progressive OR secondary) AND (intracranial OR intracerebral) AND (hemorrhage OR hematoma)) OR HPC OR hemorrhagic progression of a contusion OR DTICH OR progressive hemorrhagic injury OR PHI OR PHI

#3.PLT OR platelet counts OR PT OR prothrombin time OR D-dimer OR FDP OR Fg OR INR OR international normalized ratio OR fibrinogen OR fibrin OR fibrin degradation product OR fibrinogen degradation product OR coagulation OR coagulopathy OR PTT OR partial thromboplastin time OR DIC OR disseminated intravascular coagulation

#4. #1 and #2 and #3

**Embase**

#1.head OR cranial OR cerebral OR brain OR skull OR intracranial OR intracerebral

#2.injury OR injuries OR trauma OR traumas OR traumatic OR damage OR wound OR fracture OR contusion

#3.#1 and #2

#4.delayed OR progressive OR secondary

#5.intracranial OR intracerebral

#6.hemorrhage OR hematoma

#7.#6 and #4 and #5

#8.HPC OR ‘hemorrhagic progression of a contusion’ OR DTICH OR ‘progressive hemorrhagic injury’ OR PHI OR PIH

#9.#8 or #7

#10.PLT OR ‘platelet count’ OR PT OR ‘prothrombin time’ OR ‘D-dimer’ OR FDP OR Fg OR INR OR ‘international normalized ratio’ OR fibrinogen OR fibrin OR ‘fibrin degradation product’ OR ‘fibrinogen degradation product’ OR coagulation OR coagulopathy OR PTT OR ‘partial thromboplastin time’ OR DIC OR ‘disseminated intravascular coagulation’

#11.#3 and #9 and #10

#12.#3 AND #9 AND #10 AND [english]/lim AND [1970-2014]/py

**Cochrane Library**

#1.(head or crani* or cerebr* or capitis or brain* or forebrain* or skull* or hemispher* or intra-cran* or inter-cran*) near3 (injur* or trauma* or damag* or wound* or fracture* or contusion*) :ti,ab,kw (Word variations have been searched)

#2.((delayed OR progressive OR secondary) AND (intracranial OR intracerebral) AND (hemorrhage OR hematoma)) OR HPC OR hemorrhagic progression of a contusion OR DTICH OR progressive hemorrhagic injury OR PHI OR PHI:ti,ab,kw (Word variations have been searched)

#3.PLT OR platelet counts OR PT OR prothrombin time OR D-dimer OR FDP OR Fg OR INR OR international normalized ratio OR fibrinogen OR fibrin OR fibrin degradation product OR fibrinogen degradation product OR coagulation OR coagulopathy OR PTT OR partial thromboplastin time OR DIC OR disseminated intravascular coagulation

#4. #1 and #2 and #3

*Selection Criteria*

Studies were considered eligible if they were controlled observational studies and included people suffering traumatic brain injury (TBI).They should investigate the relationship between the abnormal coagulation tests and progressive hemorrhagic injury (PHI), which is strictly defined by the definition above. Studies were excluded if they (1) provided data from case reports, reviews; (2) examined patients with pre-trauma coagulopathy or multiple injuries, burn injuries or major injuries in other body regions rather than the brain; (3) included children(<12y), because their clotting mechanism is different from that of adults’^2^; (4) provided insufficient data for pooled analysis.

*Data Extraction and Quality Assessment*

Three reviewers (D.F.Z., S.G., H.J.)extracted the data in standardized data-collection forms, and two authors (W.Z. and P.S.) assessed the study quality. The following data were abstracted: author, publication year; study design; population(categorized as American, Asian [including Japanese populations in Hawaii],and European); sample size; participants’ characteristics(age and sex); exposure; fourfold data for dichotomous data and mean with corresponding standard deviation (SD) for continuous data. The Newcastle-Ottawa Scale (NOS) was used to evaluate the methodological quality, which scored studies by the selection of the study groups, the comparability of the groups, and the ascertainment of the outcome of interest.^3^

*Statistical Analysis*

We pooled both continuous and dichotomous data extracted from pertinent articles, using the random-effects model. When the both data were available in one paper, they were pooled into different aspects of our meta-analysis. Mean difference (MD) and odds ratio (OR) were selected as the effect sizes.We used mg/Land g/L in the measurement units of D-dimer and Fgas they were more frequently reported.

Correlation coefficient rs were calculated to assess the association strength for each parameter. All of the statistics were converted into correlation coefficient r, which was selected as the effect size, following procedures described by Rosenthal et al^4^. These rs were then combined using Fisher's z transformation and were weighted by degrees of freedom (n - 3) in order to take into account the differential precision of estimate associated with larger and smaller studies. Finally, the Fisher's Zr value was converted back to r to get a weighted average effect size for each risk factor. Higher absolute value of r indicates a stronger association with PHI. For the r metric, we considered weak, moderate, strong and powerfulr values to be <0.2, 0.2-0.4, 0.4-0.6, >0.6 respectively.

Continuous and dichotomous data were extracted respectively. Unless we were able to contact researchers to obtain missing data, we relied on the information reported in the articles. In many cases, the report was unclear about factors relating to study quality, provision of actual numbers, and details of statistical analyses. Data described as median with interquartile range (25th, 75th) in two pertinent articles were overlooked instead of being transformed into mean± SD.

In the analysis of dichotomous data in which the effect sizes are non-directional, we determined the specific trend for each coagulation parameter according to the corresponding continuous data. A threshold of P<0.1 was used to decide whether heterogeneity was present.^1^In other ways, all reported probability values were 2 sided, with significance set at <0.05. Statistical heterogeneity among studies was mainly assessed by the *I^2^*statistic.^5^ For the *I^2^* metric, we considered low, moderate and high *I^2^* values to be 25%, 50%, and 75%, respectively.^5^Sensitivity analysis was conducted by excluding one study at a time to explore whether the results were driven by one large study or by a study with an extreme result.

Inter-rater reliabilities on selection of studies and quality assessment were calculated by Cohenκstatistics, with five levels of agreement, namely poor (κ=0.00–0.20), fair (κ=0.21–0.40), moderate (κ=0.41–0.60), good (κ=0.61–0.80), and very good (κ=0.81–1.00).^1,6,7^STATA software (version 12.0; Stata Corporation, College Station, TX) was used for the meta-analysis.

*Supplemental References*

1. Cochrane handbook for systematic reviews of interventions, version 5.0.1, updated March 2011. http://www.cochrane-handbook.org. Accessed August 23 2014.
2. Ignjatovic V, Mertyn E, Monagle P. The coagulation system in children: developmental and pathophysiological considerations. Seminars in thrombosis and hemostasis 2011;37:723-729.
3. The Newcastle-Ottawa Scale (NOS) for assessing the quality of nonrandomised studies in meta-analyses. http://www.ohri.ca/programs/clinical_epidemiology/oxford.asp. Accessed August 23, 2014.
4. Brewin CR, Andrews B, Valentine JD. Meta-Analysis of Risk Factors for Posttraumatic Stress Disorder in Trauma-Exposed Adults. Journal of Consulting and Clinical Psychology 2000;68:748-766.
5. Higgins JP, Thompson SG, Deeks JJ, et al. Measuring inconsistency in meta-analyses. BMJ 2003;327:557–560.
6. Landis JR, Koch GG. The measurement of observer agreement for categorical data. Biometrics 1977;33:159-174.
7. Reichenheim ME. Confidence intervals for the kappa statistic. Stata J 2004;4:421-428.
